# Shapes of Discoid Intracellular Compartments with Small Relative Volumes

**DOI:** 10.1371/journal.pone.0026824

**Published:** 2011-11-21

**Authors:** Jure Derganc, Bojan Božič, Rok Romih

**Affiliations:** 1 Institute of Biophysics, Faculty of Medicine, University of Ljubljana, Ljubljana, Slovenia; 2 Institute of Cell Biology, Faculty of Medicine, University of Ljubljana, Ljubljana, Slovenia; University of New South Wales, Australia

## Abstract

A prominent feature of many intracellular compartments is a large membrane surface area relative to their luminal volume, i.e., the small relative volume. In this study we present a theoretical analysis of discoid membrane compartments with a small relative volume and then compare the theoretical results to quantitative morphological assessment of fusiform vesicles in urinary bladder umbrella cells. Specifically, we employ three established extensions of the standard approach to lipid membrane shape calculation and determine the shapes that could be expected according to three scenarios of membrane shaping: membrane adhesion in the central discoid part, curvature driven lateral segregation of membrane constituents, and existence of stiffer membrane regions, e.g., support by protein scaffolds. The main characteristics of each scenario are analyzed. The results indicate that even though all three scenarios can lead to similar shapes, there are values of model parameters that yield qualitatively distinctive shapes. Consequently, a distinctive shape of an intracellular compartment may reveal its membrane shaping mechanism and the membrane structure. The observed shapes of fusiform vesicles fall into two qualitatively different classes, yet they are all consistent with the theoretical results and the current understanding of their structure and function.

## Introduction

**Figure 1:**
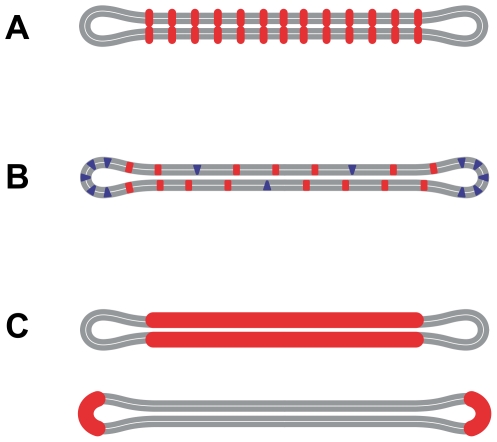
Schematic representation of three scenarios of membrane shaping. A) The discoid shape can be stabilized by adhesion between the membranes in the central part, possibly mediated by interacting luminal domains of transmembrane proteins (red). B) Weak lateral segregation of mobile membrane constituents, e.g., lipids, proteins or membrane microdomains. Segregation of wedge-shaped constituents (blue) into the rim and cylindrical constituents (red) into the flat membrane parts relaxes the bending stress in the membrane. Within the weak lateral segregation scenario, the membrane material properties vary continuously across the membrane and no distinct membrane regions with defined boundaries are observed. C) Formation of stiffer membrane regions with a defined spontaneous curvature and defined boundaries, e.g., formation of protein scaffolds that support the membrane (red). The central discoid part can be supported by a scaffold with a small spontaneous curvature (top) and the discoid rims can be supported by a highly curved scaffold (bottom).

Many intracellular compartments, such as the Golgi apparatus and the endoplasmic reticulum, exhibit flattened shapes with a large membrane surface area relative to the luminal volume, i.e., they have a small relative volume. Since the small relative volume may well be intertwined with the function of these organelles, understanding the mechanisms of their shape generation is of great interest. Different organelles often show similar morphological features despite expressing very different sets of proteins. A large part of the morphological analyses of organelles has thus focused on the mechanisms of shaping of the lipid membrane, which is their universal structural backbone [1]. It was recognized that one of the central aspects of shaping of organelles with small relative volumes is generation of high membrane curvature [2], [3] and several qualitative scenarios that could lead to the coexistence of highly curved and flat membrane have been proposed (Fig. 1).

Theoretical studies of membrane shapes have proved fruitful, yet they were primarily focused to lipid membrane compartments of relatively large relative volumes, e.g., lipid vesicles and red blood cells [4]. Systematic theoretical studies of shapes of membrane compartments with very small relative volumes, which are particularly relevant for intracellular compartments, have been rather scarce so far. Such studies would not only assist with unraveling the relation between the organelle function, their shape and the molecular structure, but also greatly facilitate the interpretation of EM micrographs of organelles, which are extremely fragile objects and very sensitive to a slightest experimental disturbance.

**Figure 2:**
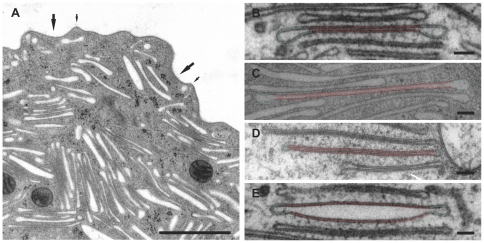
EM micrographs of fusiform vesicles in urothelial umbrella cells. A) Apical region of an umbrella cell with numerous FVs with small relative volumes. Apical plasma membrane of the cells is composed of urothelial plaques (big arrows) and hinge regions (small arrows). In B–E, the plaque regions of analyzed FVs are highlighted by light red lines, and the hinge regions by light blue lines. The relative volumes of FVs are approximately 0.1 (B–D) and 0.3 (E). The relative sizes of plaque regions with respect to the total FV surface area are: 50% (B), 63% (C), 88% (D), and 58% (E). Bars: 1 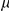
m (A), 100 nm (B–E).

A remarkable example of intracellular compartments with a small relative volume are the fusiform vesicles (FVs) of urinary bladder umbrella cells, which constitute the blood-urine barrier tissue (Fig. 2). In the central part, FVs are lined by asymmetric thickened membrane domains, called urothelial plaques, which are connected by unthickened hinge regions at the rims of vesicles [5]. Urothelial plaques are composed of hexagonally arranged 16-nm intramembrane particles, which contain four major integral proteins, uroplakins Ia, Ib, II and IIIa [6], [7]. Since urothelial plaques cover also 70–90% of the apical surface of umbrella cells, it is believed that FVs play a central role in adjusting the urothelial surface area that is needed in the course of large changes of the bladder volume during micturition cycles [8]–[10]. Atomic force microscopy measurements showed that the urothelial plaques on the apical surface have a slightly curved profile [11]. According to their appearance in transmission electron microscope, FVs were described either as biconvex discs with a fusiform profile [12], or as flattened, pancake like shapes [10].

In this study we present a systematic theoretical analysis of discoid membrane shapes with small relative volumes, and then we quantify the morphology of FVs and compare it to the theoretical results. Specifically, we build on three existing extensions of the standard approach to lipid membrane shape calculation and determine the shapes that could be expected according to three proposed scenarios of membrane shaping, i.e., membrane adhesion, curvature driven lateral segregation of membrane constituents and emergence of stiffer membrane regions with a defined spontaneous curvature. We find that both classes of observed FV shapes are consistent with the theoretical results and the current understanding of FV structure.

## Methods

### Experimental

Animals were treated in accordance with European guidelines and Slovenian legislation. The experimental protocol was approved by the Veterinary Administration at the Ministry of Agriculture, Forestry and Food of Republic of Slovenia (permit number: 34401–5/2009/4).

Three albino ICR mice (CD-1) were fed standard laboratory chow and water was available ad libitum. At the age of five weeks, they were killed with CO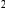, abdominal cavities were opened and bladders were cut into pieces with 2 mm diameter to fit freezing disks, and immediately frozen with liquid nitrogen at 2100 bar in a Balzers HPM 010 apparatus. Samples were freeze-substituted and embedded in Leica AFS (Leica Microsystems, Wetzlar, Germany) apparatus according to Monaghan et al. [13]: warming of samples to 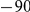 
C, freeze substitution in acetone containing 2% OsO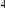 at 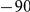 
C for 8 h, at 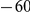 
C for 8 h and at 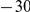 
C for 8 h. Substitution solution was changed with 100% acetone, warmed to 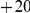 
C and embedded in Epon. From samples of each animal, two Epon blocks were selected and ultrathin sections (70 nm thick) were cut perpendicular to the urothelial surface. Sections were counterstained with lead citrate and uranyl acetate and viewed in Philips CM100 transmission electron microscope. Five micrographs of umbrella cells were taken from each section at a magnification 28500. In each micrograph 2–3 FVs were selected with longest profiles and clearly seen thickening of the limiting membrane. This yielded 50 vesicle profiles, which were analyzed with the ImageJ software (http://imagej.nih.gov/ij/). On each FV we measured the maximum length of the vesicle profile, thickness of the vesicle lumen in the central part of the vesicles, the lengths of thickened and unthickened membranes.

### Standard theoretical framework

In this section, we present the standard theoretical framework of lipid membrane shapes and discuss the challenges related to assessing shapes with small relative volumes. In the next three sections we will then employ three established extensions of the standard theory which will serve to describe the discoid shapes according to three scenarios that have been proposed for organelle membrane shaping (Fig. 1).

The shapes of closed lipid membrane compartments in equilibrium are the shapes that correspond to the minimum of the elastic energy of the membrane at given values of external parameters, e.g., the compartment volume. According to the standard model of elasticity of the lipid bilayer, the *area difference elasticity* (ADE) model [14], [15], the elastic energy of the membrane can be expressed as the sum of the membrane bending energy, Gaussian bending energy and the non-local bending energy:
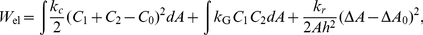
(1)where 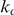 is the local mean curvature bending modulus, 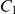 and 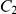 are the membrane principal curvatures, 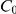 is the membrane spontaneous curvature, 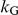 is the Gaussian bending modulus, 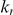 is the nonlocal bending modulus, 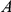 is the membrane surface area, 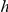 is the distance between the neutral surfaces of the two bilayer leaflets, 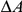 is the difference in the surface areas of the two leaflets, and 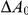 is the area difference between the leaflets when both leaflets are stress-free. The value of 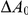 is closely related to the difference in the number of molecules in the membrane leaflets, and thus depends on the membrane lipid flip-flop and flippase activity.

Membrane elastic energy is scale invariant, i.e., it does not depend on the actual size of a compartment but rather on its relative shape [16]. Hence, the notion of a relative volume of a compartment is introduced as a dimensionless parameter describing the ratio between the actual volume of the compartment and the volume of a sphere having the same surface area as the compartment, 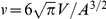. In this representation, the sphere has the largest relative volume of all shapes and it equals unity, 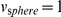. Furthermore, it has been shown that the equilibrium shapes of an axisymmetric homogeneous membrane can be calculated by solving the Euler-Lagrange equations derived from the dimensionless functional 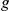 [16]:
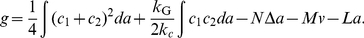
(2)Here all the dimensionless variables, written in lowercase, are rescaled with respect to a spherical membrane compartment of the given surface area. The unit of length in this representation is 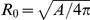, the unit of surface area is 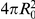 (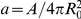), the unit of volume is 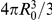 (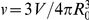), the unit of bilayer leaflet area difference is 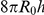 (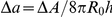) and the unit of energy is 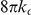. Dimensionless Lagrange multipliers (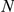, 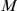 and 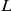) represent the relative difference between the lateral tensions of the bilayer leaflets, the relative pressure difference across the membrane, and the relative membrane lateral tension, respectively. The Lagrange multiplier 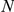 is proportional to the area difference and thus to the so called bilayer-couple effect of the lipid bilayer:
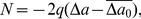
(3)where 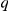 is the ratio between the bending constants 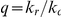, and 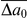 is an effective dimensionless area difference 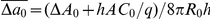. Note that the membrane spontaneous curvature 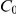 and the relaxed leaflet area difference 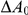 are not explicitly present in the functional 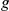 (Eq. 2), rather they influence the value of the Lagrange multiplier 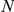 (Eq. 3). In the studies of equilibrium shapes on long time scales, a small relative difference between the lateral tensions of the bilayer leaflets is often assumed (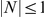), due to the membrane lipid flip-flop and due to a small relative membrane spontaneous curvature.

The Gaussian bending term is not explicitly present in the Euler-Lagrange equations, rather it is a part of the boundary conditions that arise from the variation of the functional 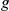. Thus, in the case of closed free homogeneous membrane compartments, the equilibrium membrane shape does not depend on the Gaussian term, and the Gaussian bending energy depends only on the compartment's topology. Moreover, there are only two independent parameters, say 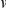 and 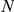, and a comprehensive phase diagram of possible ADE shapes can be represented in the 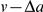 plane [16].

**Figure 3:**
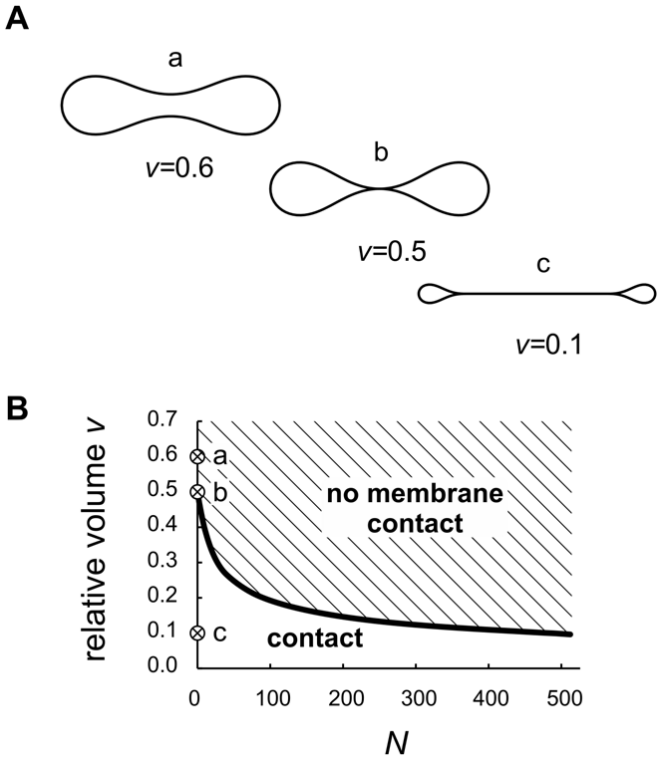
Shape features of discoid compartments with homogeneous membrane. A) Shape transformations due to a decreasing relative volume 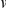 at a vanishing relative difference between the lateral tensions of the bilayer leaflets (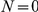). At 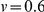, the shape is the well-known biconcave shape of a red blood cell (shape **a**). Decreasing the volume leads to a contact of the membrane at the discoid center (shape **b**, 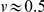) and then to an increase of the contact surface area (shape **c**, 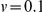). B) Phase diagram describing the values of 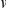 and 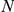 at which the membrane of a discoid compartment comes into contact with itself. The positions of the shapes **a**, **b** and **c** are presented.

The homogeneous membrane ADE model was a basis for a number of successful studies of membrane shapes with a very good agreement between theoretically calculated shapes and experimentally observed shapes of giant lipid vesicles and red blood cells, which have relative volumes 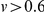 [4]. However, in the case of discoid shapes at physiological values of 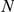, the discoid poles touch each other at lower volumes, and the membrane comes into contact (Fig. 3). At relative volumes corresponding to small relative volume membrane compartments (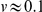) all discoid shapes have a large contact surface area. Moreover, the flattened discoid shapes with adjoining central parts are not stable, but tend to wrap up into a cup-like shape, where the surface area of the highly curved rim is minimized [17]. Also, as it has been shown in the case of starfish vesicles, the energy differences between different possible shapes at small relative volumes are very small, with no clear global energy minimum [18].

### Adhesion between membranes in the central discoid part

Adhesion between flat membrane regions has been proposed as one of the possible stabilizing mechanisms for the flattened compartment geometry in the Golgi [19] and ER [1] (Fig. 1A). In general, the membrane adhesion can result from various long range attraction forces, e.g. electrostatic or Van den Walls forces [20], as well as from molecular bridging mediated by adhesion molecules, e.g. CLIMP-63 in the ER [1]. In the first approximation, the adhesion can be modeled as an effective contact attraction. Thus, the two adhering membranes are at a constant distance and the adhesion energy is proportional to the adhesion constant 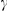:
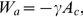
(4)where 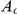 is the contact surface area (the surface area of membrane in contact is twice 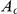).

Minimization of the total energy, 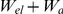, leads to two coupled sets of the standard ADE Euler-Lagrange equations, one for the free membrane in the discoid rim and the other for the adhered membrane in the central discoid part [21]. In the latter set of equations, the Lagrange multiplier 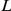 is changed to 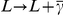, where 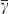 is the relative adhesion constant,
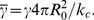
(5)The two sets of equations are coupled via an additional boundary condition imposing a discontinuous jump in the membrane curvature in the meridian direction along the line of adhesion contact: 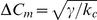 [21]–[23]. Also, there is an additional boundary condition for the radial force, resulting from a fixed distance between the adhered membranes. The standard ADE model extended by membrane adhesion has only two additional parameters: the distance between the adhered membranes, which is defined by the size of adhesion molecules, and the relative adhesion constant 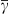.

### Weak curvature driven lateral segregation of membrane constituents

The coexistence of a highly curved membrane in the rim and a relatively flat membrane in the central discoid part can be stabilized by a nonhomogeneous distribution of membrane constituents. For example, segregation of conical molecules into the rim and cylindrical molecules into the central membrane regions leads to a local spontaneous membrane curvature that matches the actual membrane curvature and thus relaxes the bending stresses in the membrane (Fig. 1B). In the case of weak lateral segregation, the membrane composition varies continuously across the membrane and so do all the membrane material properties [24], i.e., the membrane bending constants 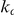 and 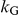, and the membrane spontaneous curvature 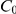 (the relaxed leaflet area difference 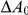 is an integral rather than local membrane property and does not depend on the local membrane composition).

Here, we will focus on the dependence of the local membrane spontaneous curvature on the intrinsic shape of membrane constituents, which can vary considerably among different lipid species [3]. In such a case, the standard ADE model has to be extended by assuming the coupling between the membrane composition and its spontaneous curvature, and by taking into account the free energy of mixing of the constituents [24]. By simplifying the problem to binary mixing, the latter can be described by the Flory-Huggins term for mixing of constituents which have different sizes:
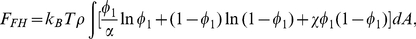
(6)where 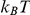 is the thermal energy, 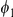 is the local surface area fraction occupied by the first species (for the second species, 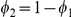), 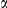 is the ratio between the molecular surface areas of the two species 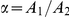, where 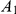 and 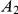 are surface areas of one molecule of the two species, respectively, and 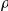 is the molecular surface density of the molecules of the second species, 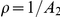. Parameter 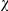 is the standard Flory interaction parameter. For regular solutions, where both species have the same surface area (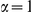), the system reaches the spinodal decomposition (e.g., spontaneous phase separation) at 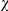 equal to 2.

The coupling between the membrane composition and its spontaneous curvature can be described as 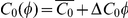, where 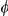 is the deviation of the local area fraction 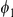 from its homogeneous value 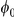, 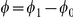, and 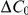 is the coupling constant, related to the intrinsic shapes of membrane constituents [24].

A seminal study of the curvature driven segregation [25] showed that in the case of binary mixing and small deviations in the local membrane concentration, the free energy of the system can be mapped onto the standard ADE model with a pronounced bilayer couple effect (for details see Supplementary Text S1). Hence, the equilibrium shapes can be calculated from the standard ADE dimensionless functional 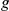 (Eq. 2) by using large values of the Lagrange multiplier 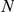. In the case of the homogeneous ADE model, such large values of 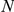 are not considered physiological as they are related to an extremely large effective area difference (Eq. 3).

Note that although no additional parameters are needed to describe the membrane shapes within the weak lateral segregation scenario, the calculation of the shapes with large 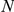 requires a careful numerical approach, with an expansion of shape equations around the discoid poles [16].

### Membrane composed of distinct regions

The third scenario addressed in the present analysis involves membranes that are composed of distinct regions with markedly different mechanical properties and well-defined boundaries. For example, large proteins accumulating in certain regions of the membrane may act as a protein scaffold and impose their intrinsic curvature to the membrane [2], [3], e.g., large flat protein scaffolds may support the central discoid part, while curved protein scaffolds may support the curved rim (Fig. 1C). In general, the proteins can be intrinsic membrane proteins, e.g., uroplakins in the fusiform vesicles [26], or externally bound to the membrane, e.g. proteins with BAR domains [27]. A similar situation may emerge if a macroscopic phase separation occurs within the membrane, e.g., if one part of the membrane is in the liquid ordered phase and the other in the liquid disordered phase.

Within this scenario, the discoid membrane compartments can be described by two connected ADE regions, one being stiffer with a defined spontaneous curvature, and the other being normal with a vanishing spontaneous curvature. The minimization of the total elastic energy of the membrane then leads to two coupled sets of the standard ADE Euler-Lagrange equations with separate sets of ADE parameters. It turns out, that the shape is not affected by the absolute stiffness of the two regions but rather by the relative stiffness of the stiffer one 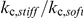. Additionally, the coupling between the two membrane regions involves the line tension between the two membrane regions and the Gaussian bending constants [28], [29].

Clearly, the parameter space in this case is rather large, and therefore the present analysis will focus to the effects of the three most relevant parameters: the relative stiffness of the stiffer membrane region, its relative size and its spontaneous curvature. All other parameters will be held in their plausible range, e.g., the value of the Gaussian bending constant will be 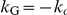 [30], line tension will be zero, and spontaneous curvatures of the stiffer regions will correspond to the actual curvatures of those regions. Two qualitatively different scenarios are considered. First, the stiffer membrane has a small spontaneous curvature and supports the flat discoid sides, and second, the stiffer membrane has a large spontaneous curvature and supports the discoid rim.

The analysis presented does not take into account two properties that generally play a role in the mechanics of protein scaffolding and multicomponent membranes. First, a possible shear rigidity of the protein scaffold has been neglected. It can be shown theoretically that the shear rigidity does not influence the equilibrium membrane shape in the limit of small deformations of a nearly flat scaffold (see Supplementary Text S1). Also, it is known that the membrane spectrin skeleton, which contributes the shear rigidity to the erythrocyte membrane, does not significantly affect the equilibrium discocyte shape of the erythrocyte [4]. Still, the possible role of the shear rigidity in the highly curved rims remains to be analyzed. Second, the study neglects the effects of a line tension between the two membrane regions. A line tension between the two membrane regions could lead to a) a contact angle between the membrane regions [29], [31], [32], and b) to instability of the discoid shapes caused by a tendency to decrease the length of the contact line between the two regions [33]. If these effects are not present in a membrane compartment, the omission of the line tension is justified.

## Results

### Shape of fusiform vesicles

The superficial layer of urothelium contained large umbrella cells. They were covered with the apical plasma membrane, formed by concave asymmetrically thickened membrane regions (urothelial plaques) and slightly raised unthickened membrane (hinge) regions. The cytoplasm of umbrella cells contained numerous flattened FVs (Fig. 2A). We analyzed the shape of 50 clearly distinguishable FVs, which were cut approximately through their perimeter. FVs had two opposing urothelial plaques and slightly dilated hinge regions at rims. The plaques were 12 nm thick and had diameters 680–850 nm in FVs that measured 850–1100 nm along their long axis (Table 1). Along the urothelial plaque, the thickness of FV was 30 nm on average. The rims had the radius between 13 and 42 nm. The length of the hinge region varied from 214 nm to 433 nm. Some variation in the vesicle profiles was observed. In vesicles with relative volumes close to 0.1, the space between two plaques was narrow and measured 6
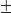
2 nm (Fig. 2B–D). Vesicles with relative volumes larger than 0.1 had a more convex shape of plaques (Fig. 2E).

**Table 1:** Data from measurements of fusiform vesicles.

	maxlength	lumenthickness	thickenedmembranelength	plaquearea^1^	unthickenedmembranelength	hingeradius
AVERAGE	960*	6	765	0.46**	308	26
ST. DEV.	112	2	84	0.10	49	7

1 plaque surface area is calculated as plaque being a round structure,

*all measures are in nm, except areas,

**which are in 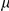
m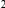.

### Calculated shapes

In order to obtain an insight into the variety of possible shapes at small relative volumes, we will focus to the discoid shapes calculated at the relative volume 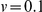 and within representative parameter ranges. The shapes calculated according to the three proposed scenarios will be compared to the shape obtained by the standard homogeneous ADE model (shape **c**, Fig. 3A). Typical values of geometrical parameters will correspond to a typical FV with a surface area of 1.7 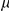
m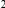, the rim curvature radius of 26 nm and the plaque region comprising approximately 60% of the total membrane surface area. The spontaneous curvature of the plaques, which can be observed on the apical plasma membrane in Fig. 2A, was not measured in this study. However, the cross-sectional curvature of the plaques was previously measured to be approximately 1/(460 nm) [11], which yields the spontaneous curvature of the plaques to be on order of 1/(230 nm). Thus, in the dimensionless relative notation, the unit of length 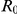 will be approximately 365 nm, the relative curvature in the rim approximately 15 and the relative spontaneous curvature of the plaque on the order of unity.

**Figure 4:**
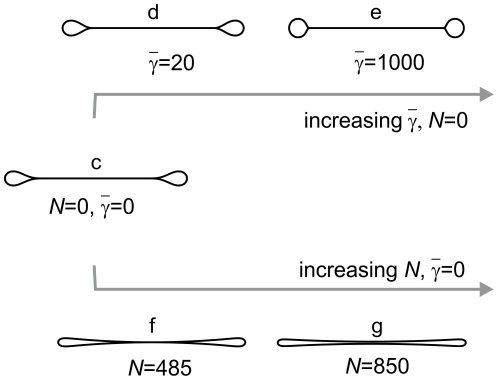
Shape changes due to adhesion in the central discoid part (top, shapes d and e) and lateral segregation of membrane constituents (bottom, shapes f and g). Shape **c** is the shape from the standard homogeneous model (Fig. 3A). The adhesion strength is represented by the relative adhesion constant 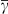; an increase of 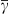 increases the contact surface area. The impact of the lateral segregation is described by parameter 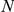; an increase in 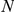 corresponds to more convex shapes with a smaller contact surface area. The relative volume of all shapes is 0.1.

We start by examining the effects of adhesion and curvature driven lateral segregation, as these two models are straightforward extensions of the standard homogeneous ADE model. Fig. 4 compares the effects of adhesion (top, shapes **d** and **e**) and lateral segregation (bottom, **f** and **g**) on the standard ADE shape (shape **c**).

The main effect of adhesion (Fig. 4, shapes **d** and **e**) is an increase in the membrane contact surface area. While the surface area of membrane in contact within the standard ADE model in absence of intermembrane adhesion is approximately 32% of the total membrane surface area, it reaches approximately 49% in the limit of strong adhesion. Correspondingly, an increasing adhesion makes the shape of the rim more and more round. The maximal contact surface area could increase further with a decreased relative volume. Fig. 4 shows the shapes with zero gap between the adhering membranes, yet the qualitative picture does not alter even if the gap is set to a finite distance (e.g. to 10 nm), which could be the case if the adhesion is mediated by molecular bridging.

The effect of weak lateral segregation of membrane constituents is presented in the bottom part of Fig. 4 (shapes **f** and **g**). Weak lateral segregation effectively increases the bilayer couple effect in the membrane, i.e., increases the effective difference in lateral tensions between the membrane leaflets, described by the dimensionless parameter 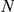 (Eq. 3). Larger values of 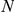 correspond to more convex discoid shapes with a smaller contact surface area in the central part. The separation of the two sides in the central part occurs at 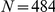 (see Fig. 3B, and shape **f** in Fig. 4). A further increase of 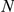 corresponds to even more convex shapes, yet the membranes in the center remain relatively close together even at very large values of 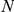; for example, even at 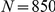, the distance between the two membranes in the center is 0.03 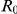, i.e., approximately 10 nm (shape **g**).

**Figure 5:**
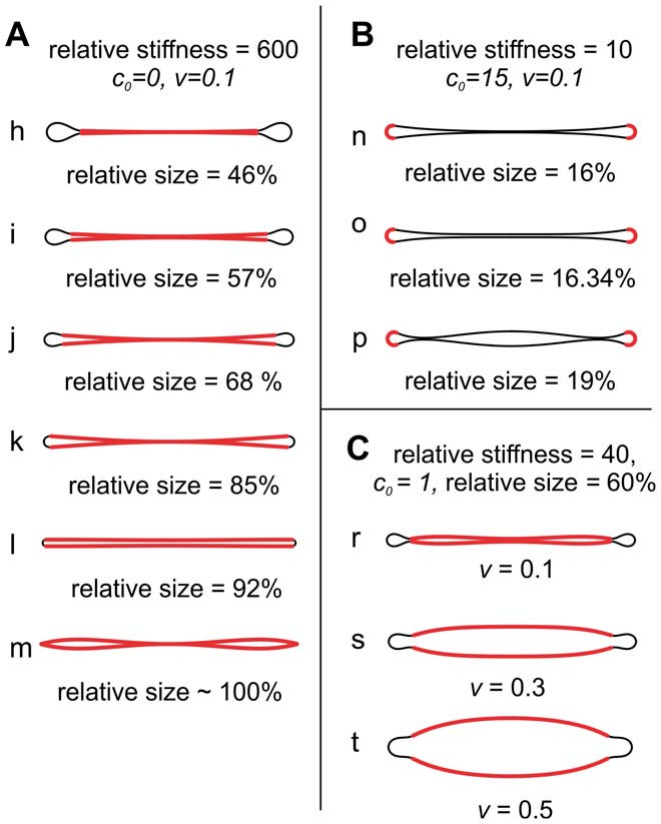
Examples of shapes with two distinct membrane regions. The stiffer membrane regions are presented by thick red lines and the soft membrane regions by thin black lines. The relative stiffness (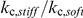), the spontaneous curvature (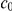) and the relative size of the stiffer regions, and the relative volume of the compartment (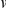) are denoted. The spontaneous curvature of the soft region is zero. A) Effects of an increasing size of a very stiff region with 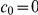 in the central discoid part. B) Effects of an increasing size of a curved (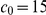) stiffer region supporting the discoid rim. C) Effects of an increasing relative volume in the case corresponding to the observed FV: the stiffer region is in the central discoid part, it occupies 60% of the total membrane surface area and has 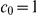. The relative stiffness of the stiffer region is chosen to be 40.

**Figure 6:**
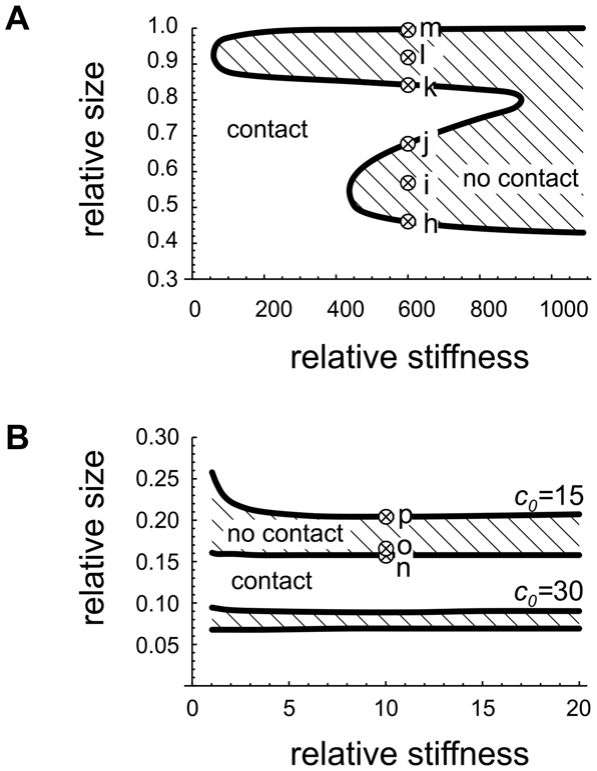
Phase diagrams showing the regions where the compartment's membrane is not in self-contact (dashed regions). The calculated dependence on the relative stiffness and the relative size of the stiffer membrane region is presented. The diagrams correspond to the shapes presented in Fig. 5 A and B. A) The stiffer membrane region has zero spontaneous curvature and supports the central discoid parts, shapes **h** through **m** in Fig. 5A. B) The stiffer region is curved and supports the discoid rim, shapes **n** through **p** in Fig. 5B. The diagram is given for two values of the spontaneous curvature in the rim (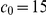 and 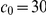). The crossed circles correspond to the shapes presented in Fig. 5.

According to the third proposed scenario, the discoid shapes are stabilized by regions of stiffer membrane with a defined spontaneous curvature. The results will focus to the three parameters: the relative stiffness, the relative size and the spontaneous curvature of the stiffer membrane region. Some of the typical calculated shapes are presented in Fig. 5. Not surprisingly, a much wider parameter space results in a much more complicated shape behavior. To assist the orientation in the parameter space, Fig. 6 shows the calculated values of the relative stiffness and size of the stiffer region at which the membrane in the central part is not in contact.

The impact of a very stiff membrane region in the central discoid part is presented in Figs. 5A (shapes **h** through **m**) and 5A. If the stiff region is small, the membranes in the central part are in contact, just as with the homogeneous membrane. As the stiff region grows in size, the central discoid part first separates when the stiffer region occupies 46% of the total membrane, comes into contact again at 68%, only to detach once more at 85%. Finally, as the stiff membrane region grows towards 100% of the total membrane, the membrane comes back in contact and the shape approaches the standard homogeneous ADE shape. The point of contact is always in the discoid center. Fig. 6A shows that the behavior is less complicated at smaller stiffness. If the stiffer membrane region is less than 400 times stiffer than the soft region, the membranes lose contact only at relative sizes larger than 80%. If the stiffer membrane region is less than 60 times stiffer than the soft region, the membrane in the central part remains in contact at all sizes of the stiff region. At a small relative size and stiffness of the stiffer region, the shapes have a large contact area and resemble the shape of a homogeneous membrane.

The effects of a stiffer membrane region with a large spontaneous curvature supporting the discoid rim are presented in Fig. 5B and 6B (shapes **n** through **p**). As the size of the stiffer region increases, the central discoid sides first separate at the discoid center (shape **n**) and then come into contact again away from the center (shape **p**). In other words, the curved rim forces the central discoid part to undulate. Interestingly, Fig. 6B shows that in order to have a marked effect on the shape, the stiffer region does not need to be any stiffer at all, it only needs to have a large enough spontaneous curvature (at this relative volume, it has to be larger than approximately 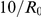, calculation not shown). Also, the larger the spontaneous curvature of the stiffer membrane, the smaller region it needs to occupy. A closer inspection of the shapes reveals that the actual curvature in the rim is up to 10% smaller than the spontaneous curvature imposed by the stiffer region.

Finally, Fig. 5C shows the shapes obtained if the stiff region is in the central discoid part and has a small but non-vanishing spontaneous curvature, a case relevant for FVs. Here, the undulation in the central discoid part is noticeable again: the central discoid part comes in contact in its center due to the small relative volume, and, in addition, it comes in contact near the rim due to its spontaneous curvature. The biconvex “fusiform” shape can only be obtained at larger relative volumes.

## Discussion

The aim of this work was to theoretically analyze three scenarios for membrane shaping in discoid intracellular compartments at small relative volumes [1]–[3] (i.e., membrane adhesion in the central discoid part, weak lateral segregation of membrane constituents and formation of distinct membrane regions) and to explore the implications of the theory on current understanding of the shape and structure of fusiform vesicles (FVs) in urinary bladder umbrella cells.

The first conclusion of the theoretical analysis is that all three scenarios of membrane shaping can lead to qualitatively similar shapes with a flattened central part and a drop-like cross-section at the rim, a shape comparable to the shape theoretically associated with simple homogeneous membrane (shape **c** in Figs. 3 and 4). This type of shape has been indeed observed in cellular systems, e.g., in the Golgi [34], in the discs in the eye rod outer segment [35], and FVs [36]. This shape can be therefore regarded as a generic small relative volume discoid shape resulting from the membrane tendency to minimize its overall bending at a given small relative volume. In addition, this configuration can be further enhanced by a variety of nonspecific interactions which emerge between two nearby membranes [20]. As a consequence, if a intracellular compartment has a flattened central part with adjacent membranes and a drop-like rim, its shape cannot straightforwardly reveal the shaping mechanisms or the material properties of its membrane.

On the other hand, at certain values of model parameters the three scenarios can also yield different shapes. In these cases, the shape of the compartment can in fact indicate its underlying shaping mechanism. For example, with an increasing adhesion between the membranes in the central part, the rim becomes more and more round (shape **e** in Fig. 4), making it possible to estimate the interaction strength between the membranes. The value of the adhesion strength corresponding to the calculated shapes depends on the actual size of the compartment and its membrane bending constant (Eq. 5). Taking that the membrane bending constant is within typical values of lipid membranes (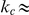
20 kT) and the size of the compartment compares to the size of a FV, the strong adhesion limit (shape **e**) corresponds to an adhesion strength of approximately 10000 kT per 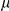
m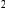. If the adhesion is mediated by molecular bridges with the binding energy of 5 kT, this leads to approximately 2000 adhesion molecules per 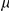
m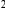.

Curvature driven lateral segregation of membrane constituents generally drives the membrane into more convex shapes (shapes **f** and **g** in Fig. 4). While the actual extent of this kind of segregation in cellular organelles is not yet characterized, theoretical analyses and experimental studies of synthetic lipid bilayers have shown several interesting aspects of this phenomenon. First, the larger the membrane constituents the more easily they segregate, which indicates one of the possible advantages of membrane microdomain formation [37]. Second, an extensive segregation can take place only in the presence of large intermolecular interactions in the membrane, i.e., in the proximity of the phase segregation of the membrane constituents [38], [39]. A closer inspection of the model in this study reveals that large values of the parameter 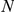 (Eq. 3) and therefore noticeable effect on the shape can be realized even when the local membrane composition does not vary considerably across the membrane, provided there is strong coupling between the membrane composition and its spontaneous curvature (Supplementary Text S1).

According to the third scenario, the membrane shaping is driven by the emergence of distinct membrane regions, which can in general result from a variety of mechanisms, e.g. from protein scaffolding or from large scale lipid phase separation. Theoretical analysis presented shows that this scenario can lead to a wide range of different membrane shapes, depending notably on the relative size and stiffness of the membrane regions and their spontaneous curvature. Characteristically, certain values of these parameters result in shapes where the membrane does not touch itself in the discoid center but rather closer to the rim, e.g., shape **p** in Fig. 5B. Also, there is a strong dependence of the shape on the relative size of the membrane regions: in Fig. 5B the stiffer region in the rim grows from only from 16% to 19% of the overall membrane surface area, and consequently the shape changes dramatically from shape **o** to shape **r**. Thus, unless the key parameters are regulated by the cell, this shaping mechanism can result in compartments with many qualitatively different shapes.

The values of model parameters for the third scenario in biological membranes are yet to be determined. While it is known that the liquid ordered lipid phase is several times stiffer than a liquid disordered phase [30], [32], [38], we are not aware of quantitative data on membrane stiffening by protein scaffolds. The theoretical analysis in this study clearly shows that spontaneous curvature of protein scaffolds supporting the rim may be even more important than their relative stiffness (Fig. 6). For example, the spontaneous curvature of the BAR domain was found to be on the order of 1/(10 nm) [27], and therefore these proteins could easily provide the curvature needed to support the curved rim regardless of their possible stiffening effect on the membrane. We found that the actual curvature of the membrane in the highly curved rim can differ from its spontaneous curvature up to approximately 10% (the softer the region, the larger the difference), which indicates that a protein scaffold supporting the curved rim may well be under the strain imposed by the membrane.

The theoretical analysis has focused on the calculation of the discoid shapes and did not explore the global energy landscape of all possible shapes and did not consider cup-like shapes. In effect, a simple argument suggests that the discoid shapes calculated according to the adhesion scenario are not necessarily the global minima: in the limit of small adhesion they would wrap up into cup shapes (as within the standard homogeneous membrane model), and in the limit of strong adhesion, the lumen of the compartment would become spherical with all the excess membrane adhered and wrapped up. In addition, one can expect small energy differences between different shapes at small relative volumes [18]. As a consequence, the existence of other non-discoid shape classes cannot be ruled out. In fact, cup-like shapes have been indeed observed in some vesicles of urothelial cells [40] and also in the Golgi [41]. Thus, for a critical assessment of the global stability of shapes of intracellular compartments, all the model parameters should be carefully identified as well as possible interaction with other cellular structures nearby.

The shape of FVs in umbrella cells can be best studied by transmission electron microscopy. We aimed to prepare mouse urothelium in a way that preserved the ultrastructure closest to its native state. Therefore we applied high pressure freezing for tissue fixation, which immobilized cellular structures within a few milliseconds [42]. Transmission electron microscopy images show that the limiting membrane of FVs is composed of two distinct regions, the asymmetrically thickened region in the central region and the unthickened membrane in the rims. The first region corresponds to urothelial plaques while the other to the hinge. Previous studies indicate that the curvature of urothelial plaques in the apical plasma membrane is on the order of 1/(250 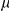
m), with the curvature center on the urine side [11]. A majority of large FVs with a small relative volume, however, has a flattened appearance, with the two opposing plaques nearly parallel to each other. As the plaque is approximately twice as thick as bare membrane, it can be expected that its stiffness is at least an order of magnitude larger than the stiffness of bare membrane.

The shapes of FVs with a small relative volume (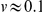) fall into the “generic” shape category, i.e., they have adjoining membranes in the central part and a drop like rim (Fig. 2B–D). Generally these shapes cannot be linked to a particular membrane shaping mechanism. On the other hand, the shapes of FVs with larger relative volumes show the distinct fusiform profile (Fig. 2E). However, both classes of the observed FV shapes are consistent with the model assuming that the plaque regions form a scaffold with a defined spontaneous curvature. Specifically, at larger relative volumes the plaques retain their spontaneous curvature and FVs exhibit the characteristic fusiform biconvex shape (shapes **s** and **t** in Fig. 5C). In contrast, if the FV volume is sufficiently small, the two opposing plaques are being pushed one against the other and flattened out (shape **r** in Fig. 5C).

Within the experimental resolution in this study, the observed shapes of FVs at small relative volumes correspond to the calculated shapes obtained with a wide range of values of the model parameters (e.g., compare shapes **i** and **r** in Fig. 5). Consequently, the study does not allow for a quantitative assessment of FV membrane material properties, such as that performed on synthetic giant lipid vesicles [29], [31], [32]. In addition, in order to describe properly the shape with a flattened plaque, the theoretical modeling has to be extended beyond the analysis presented in this work, i.e., by combining the adhesion and the protein scaffold scenarios into one computational model [43]. On the other hand, the analysis indicates a promising direction for future research, i.e., the largest differences between different shaping mechanisms and a better resolution for fitting the model parameters to the observed shapes can be expected at varying experimental conditions. For example, if the relative volume of FVs is controllably changed at a fixed plaque relative size, one can expect a series of FV shapes similar to Fig. 5C. Likewise, an altered uroplakin expression could lead to a non-symmetrical organization of the plaque in the membrane, revealing its spontaneous curvature and relative stiffness.

Since FVs are cell compartments for the transport of membranes only, their small volume-to-surface area ratio has important biological implications. First, the urothelium as blood-urine barrier forming tissue synthesize large amounts of urothelial plaques without secretory products. In this case, FVs represent a cytoplasmic pool of membrane, which can be inserted into the apical surface [44]. Second, if FVs are retrieved from the apical surface during bladder contraction [10], their small relative volume ensures minimal internalization of toxic substances from the urine.

To conclude, the study presented is aimed at bridging the gap between the standard physical theory of membrane shapes and biology of intracellular compartments. It provides an overview of theoretical modeling of membrane shaping, and the application of the theory to a real biological system. The analysis shows good consistency between the observed shapes of fusiform vesicles, the current understanding of their molecular structure, their function and the theory. In addition, the study can serve as a basis for studies of other systems, e.g., the discs in the eye rod outer segment, where protein spacers seemingly mediating inter-membrane interaction have been observed [35].

## Supporting Information

Text S1 Supplementary Text S1 provides a detailed discussion on combining the ADE model of membrane elasticity with Flory-Huggins free energy of mixing and an analysis of shear rigidity in nearly flat, axisymmetric membranes.
(PDF)
