# Supplementary material for: Shapes of Discoid Intracellular Compartments with Small Relative Volumes
Source: PLoS One. 2011 Nov 21;6(11):e26824. doi: 10.1371/journal.pone.0026824 (PMC3221666; doi:10.1371/journal.pone.0026824)
Supplement: Text S1 — Supplementary Text S1 provides a detailed discussion on combining the ADE model of membrane elasticity with Flory-Huggins free energy of mixing and an analysis of shear rigidity in nearly flat, axisymmetric membranes. (PDF) [file pone.0026824.s001.pdf]

Supplementary Text S1

Combining ADE model with Flory-Huggins free energy of mixing

In his seminal study [1], Seifert showed how to incorporate weak curvature driven lateral segregation into the standard ADE framework [2, 3]. Here we will apply this remapping to the lateral segregation described by the Flory-Huggins free energy term and express the results in the standard dimensionless notation [2]. We show that the remapping can introduce a large effective difference between the lateral tensions of the bilayer leaflets even in absence of extensive lateral segregation.

In binary mixtures, the Flory-Huggins free energy of mixing can be written as (Eq. 6 in the main text):

$$F_{FH} = k_B T \rho \int [\frac{\phi_1}{\alpha} \ln \phi_1 + (1-\phi_1)\ln(1-\phi_1) + \chi\phi_1(1-\phi_1)] dA \ , \tag{S1}$$

where $k_B T$ is the thermal energy, ϕ_1 is the local surface area fraction occupied by the first species (for the second species, $\phi_2 = 1 - \phi_1$), α is the ratio between the molecular surface areas of the two species $\alpha = A_1/A_2$, where A_1 and A_2 are surface areas of one molecule of the two species, respectively, and ρ is the molecular surface density of the molecules of the second species, $\rho = 1/A_2$. Parameter χ is the standard Flory interaction parameter.

For small deviations of the local membrane composition, the dimensionless Flory-Huggins free energy of mixing can be approximated by the second term in the series expansion of the free energy around the homogeneous composition ϕ_0 [1]:

$$f_{FH} = \frac{1}{4}\int \overline{f_{FH}} da \approx \frac{1}{4}\int \Theta \phi^2 da, \tag{S2}$$

where f_{FH} is the dimensionless form of the Flory-Huggins energy, $f_{FH} = F_{FH}/8\pi k_c$, ϕ is the deviation of the local area fraction ϕ_1 from its homogeneous value ϕ_0, $\phi = \phi_1 - \phi_0$, and Θ is the second derivative of the dimensionless free energy at the homogeneous composition:

$$\Theta = \frac{\partial^2 \overline{f_{FH}}}{\partial \phi_1^2}\bigg|_{\phi_1=\phi_0} = \frac{k_B T \rho R_0^2}{k_c}\left[\frac{1}{\alpha\phi_0} + \frac{1}{(1-\phi_0)} - 2\chi\right] \ . \tag{S3}$$

As Θ approaches zero, the system approaches the spinodal decomposition. For regular solutions, where both species have the same surface areas, the critical value of the Flory interaction parameter χ is equal to 2.

The dimensionless total free energy of the system is the sum of the membrane elastic energy and the free energy of mixing:

$$f = w_{\text{el}} + f_{FH} = \frac{1}{4}\int (c_1 + c_2 - c_0(\phi))^2 da + q(\Delta a - \Delta a_0)^2 + \frac{1}{4}\int \Theta\phi^2 da \ , \tag{S4}$$

where the local spontaneous curvature is coupled to the local composition $c_0(\phi) = \overline{c_0} + \Delta c_0 \phi$, with $\overline{c_0}$ the spontaneous curvature of homogeneous membrane and Δc_0 the coupling constant, related to the intrinsic spontaneous curvatures of membrane constituents . It can be easily verified that minimizing this free energy leads to a direct solution for the local membrane composition [1]:

$$\phi = \frac{\Delta c_0}{\Theta + \Delta c_0^2}\left(c_1 + c_2 - 2\Delta a\right) \ . \tag{S5}$$

Putting the solution for ϕ into the expression for the free energy (Eq. S4), the free energy can be rewritten into the standard ADE form:

$$\widehat{f} = \frac{f}{\widehat{\kappa}} = \frac{1}{4}\int (c_1 + c_2)^2 da + \widehat{q}(\Delta a - \widehat{\Delta a_0})^2 + const.\ , \tag{S6}$$

where $\widehat{\kappa}$ is the effective bending constant

$$\widehat{\kappa} = \left(1 + \frac{\Delta c_0^2}{\Theta}\right)^{-1} \tag{S7}$$

and the remapped ADE parameters are

$$\widehat{q} = \frac{1 + q - \widehat{\kappa}}{\widehat{\kappa}} \qquad \text{and} \qquad \widehat{\Delta a_0} = \frac{q}{1 + q - \widehat{\kappa}}(\Delta a_0 + \frac{c_0}{2q})\ . \tag{S8}$$

The remapped effective difference between the lateral tensions of the bilayer leaflets reads

$$\widehat{N} = -2\widehat{q}(\Delta a - \widehat{\Delta a_0}). \tag{S9}$$

Stationary shapes of the membrane with lateral segregation are clearly the same as the stationary shapes of the homogeneous membrane with pronounced bilayer-couple effects. As the system approaches spinodal decomposition ($\Theta \to 0$), the effective bending constant ($\widehat{\kappa}$) approaches zero (Eq. S7) and the relative non-local bending constant ($\widehat{q}$) approaches infinity (Eq. S8), which drives the system into the bilayer-couple model limit [4].

Note that the extent of the lateral segregation (Eq. S5) does not depend directly on the effective bending constant ($\widehat{\kappa}$) and the relative non-local bending constant ($\widehat{q}$). It thus turns out that, at sufficiently large Δc_0, the system can approach the bilayer-couple limit even without extensive lateral segregation, i.e., in the limit of weak lateral segregation where the deviations of the local membrane composition are small. For example, at $\Delta c_0 = 300$ and $\Theta = 1000$, the maximal local deviations of the membrane composition (Eq. S5) are less than 10%, yet the effective bending constant (Eq. S8) is 0.01 and the effective Lagrange multiplier $\widehat{N}$ (Eq. S9) is on the order of several thousand.

Shear rigidity in nearly flat, axisymmetric membranes

Material properties of stiffer membrane regions that emerge due to protein scaffolding may in general differ from the properties of a pure lipid membrane which is considered as a two-dimensional liquid. Therefore, apart from the standard ADE energy terms, additional elastic energy terms may become relevant. In general, the additional energy can be described as a sum of the local stretching and shear energies [5]:

$$F = \frac{K_\alpha}{2}\int (\lambda_1\lambda_2 - 1)^2 dA + K_\mu \int \frac{(\lambda_1 - \lambda_2)^2}{2\lambda_1\lambda_2} dA\ , \tag{S10}$$

where λ_1 and λ_2 are the local principal stretches, K_α and K_μ are the elastic moduli for stretching and shear, respectively, and the integrals extend over the undeformed shape with the surface area A_{scaffold} [6,7]. Eq. S10 measures the elastic-energy cost of the protein scaffold that depends both on the relaxed shape of the scaffold and on the way it is actually distributed over the membrane surface. To apply Eq. S10, it is necessary to specify a nominal relaxed shape of the scaffold for which the strain energy vanishes. Here, we chose the shape with constant curvatures and homogeneous initial conformation of the protein scaffold.

Axisymmetric membrane can be parameterized with the arch-length. For small deformations and nearly flat membrane in the central discoid part, we can expand the shape of the membrane in small deviations from the initial shape

$$r = r_0 + \epsilon_1 s_0 + \epsilon_2 s_0^2 + ... \tag{S11}$$

and

$$s = s_0 + \xi_1 s_0 + \xi_2 s_0^2 + ... \tag{S12}$$

where r_0 and r are the distances between the symmetry axis and a part of the protein scaffold before and after the deformation, respectively, and s_0 and s are the arch-lengths of the same part of the scaffold before and after the deformation, respectively. The corresponding series coefficients are denoted as ϵ_i and ξ_i. By taking into consideration that close to the symmetry axis the radius in nearly flat regions is proportional to the arch-length, we obtain

$$\epsilon_1 = \xi_1\,. \tag{S13}$$

For axisymmetric case the local principal stretches are expressed by the equations $\lambda_1 = r/r_0$ and $\lambda_2 = ds/ds_0$ [7]. Inserting Eqs. S11 and S12 into the expressions for the energy of the scaffold (Eq. S10), and considering Eq. S13, we obtain the energy of the scaffold written in terms of ϵ_i and ξ_i. By neglecting the terms with $\epsilon_{i+1}s_0$ and $\xi_{i+1}s_0$ in comparison with the terms with ϵ_i and ξ_i, the energy of a nearly flat scaffold in the limit of small deformations is written as

$$F = 2K_\alpha \epsilon_1^2 A_{\mathrm{scaffold}} \tag{S14}$$

It can be seen that at small shape deviations the effects of the shear energy are negligible. Moreover, the effect of the deformation of the scaffold is proportional to its area, and consequently, the scaffold effectively changes the lateral tension in the membrane.

References

1. Seifert U (1993) Curvature-induced lateral phase segregation in two-component vesicles. Phys Rev Lett 70: 1335–1338.

2. Božič B, Svetina S, Žekš B, Waugh RE (1992) Role of lamellar membrane structure in tether formation from bilayer vesicles. Biophys J 61: 963-973.

3. Miao L, Seifert U, Wortis M, Döbereiner HG (1994) Budding transitions of fluid-bilayer vesicles - the effect of area-difference elasticity. Phys Rev E 49: 5389-5407.

4. Seifert U (1997) Configurations of fluid membranes and vesicles. Adv Phys 46: 13–137.

5. Landau LD, Lifshitz EM (1976) Mechanics, Third Edition: Volume 1 (Course of Theoretical Physics). Butterworth-Heinemanns.

6. Evans EA, Skalak R (1980) Mechanics And Thermodynamics Of Biomembranes. CRC Press.

7. Mukhopadhyay R, Lim GHW, Wortis M (2002) Echinocyte shapes: Bending, stretching, and shear determine spicule shape and spacing. Biophys J 82: 1756 - 1772.
